# Caudate Nucleus Volume Mediates the Link between Cardiorespiratory Fitness and Cognitive Flexibility in Older Adults

**DOI:** 10.1155/2012/939285

**Published:** 2012-07-31

**Authors:** Timothy D. Verstynen, Brighid Lynch, Destiny L. Miller, Michelle W. Voss, Ruchika Shaurya Prakash, Laura Chaddock, Chandramallika Basak, Amanda Szabo, Erin A. Olson, Thomas R. Wojcicki, Jason Fanning, Neha P. Gothe, Edward McAuley, Arthur F. Kramer, Kirk I. Erickson

**Affiliations:** ^1^Department of Psychology, Carnegie Mellon University, Pittsburgh, PA 15213, USA; ^2^Center for the Neural Basis of Cognition, Carnegie Mellon University, Pittsburgh, PA 15213, USA; ^3^Department of Psychology, University of Pittsburgh, 3107 Sennott Square, 210 South Bouquet Street, Pittsburgh, PA 15260, USA; ^4^Department of Psychology, University of Iowa, Iowa city, IA 52242, USA; ^5^Department of Psychology, The Ohio State University City, Columbus, OH 43210, USA; ^6^Department of Psychology, University of Illinois, Champaign-Urbana at Champaign, IL 61820, USA; ^7^Beckman Institute for Advanced Science and Technology, University of Illinois at Champaign-Urbana, Champaign, IL, USA; ^8^Department of Psychology, The University of Texas at Dallas, Dallas, TX 75080, USA; ^9^Department of Kinesiology and Community Health, University of Illinois, Champaign-Urbana at Champaign, IL 61820, USA

## Abstract

The basal ganglia play a central role in regulating the response selection abilities that are critical for mental flexibility. In neocortical areas, higher cardiorespiratory fitness levels are associated with increased gray matter volume, and these volumetric differences mediate enhanced cognitive performance in a variety of tasks. Here we examine whether cardiorespiratory fitness correlates with the volume of the subcortical nuclei that make up the basal ganglia and whether this relationship predicts cognitive flexibility in older adults. Structural MRI was used to determine the volume of the basal ganglia nuclei in a group of older, neurologically healthy individuals (mean age 66 years, *N* = 179). Measures of cardiorespiratory fitness (VO_2max_), cognitive flexibility (task switching), and attentional control (flanker task) were also collected. Higher fitness levels were correlated with higher accuracy rates in the Task Switching paradigm. In addition, the volume of the caudate nucleus, putamen, and globus pallidus positively correlated with Task Switching accuracy. Nested regression modeling revealed that caudate nucleus volume was a significant mediator of the relationship between cardiorespiratory fitness, and task switching performance. These findings indicate that higher cardiorespiratory fitness predicts better cognitive flexibility in older adults through greater grey matter volume in the dorsal striatum.

## 1. Introduction

Age-related cognitive decline is an unfortunate, but nearly ubiquitous, characteristic of late life that is preceded by atrophy of several brain regions including the prefrontal cortex, medial temporal lobe, and basal ganglia [1, 2]. Because of the expected increase in the proportion of adults over the age of 65 in the next forty years, it has become a major public health initiative to identify methods to prevent or reverse regional brain atrophy with the hope that this might concurrently improve cognitive performance [3]. Randomized trials of aerobic exercise have proven promising from this regard, with participation in exercise programs leading to greater prefrontal [4] and hippocampal volumes [5]. Nonrandomized longitudinal studies of physical activity [6, 7] and cross-sectional studies of cardiorespiratory fitness [8–10] have shown similar results, with more physical activity and higher fitness levels associated with greater volumes.

 Unfortunately, few studies have examined whether cardiorespiratory fitness levels in older adult humans are associated with brain areas other than the prefrontal cortex and hippocampus [4–6, 8–10]. The striatum is of particular interest because it shows relatively early and rapid age-related atrophy, [2, 11] is a critical node in motor circuitry, supports task coordination and attentional control processes, and disruption of its dopamine circuits is linked to common age-related disorders such as Parkinson's disease [12, 13]. Yet, there is emerging evidence that exercise directly influences dopamine circuitry, angiogenesis, and cell signaling cascades in the striatum. For example, rodent research has found that voluntary wheel running increases the production of dopamine and brain-derived neurotrophic factor in the dorsal striatum [14–16]. Exercise is also considered to be one of the more promising treatments for attenuating dopamine deficiency in 6-hydroxydopamine models of Parkinson's disease [17, 18]. In concert with this, exercise ameliorates motor and cognitive deficits in adults with Parkinson's disease [19, 20]. Finally, higher cardiorespiratory fitness levels are associated with larger caudate nucleus and putamen volumes in children and these volume differences were in turn associated with better performance on a measure of attentional control [21]. Yet, the possible link between fitness levels and basal ganglia structure in older adults has not been investigated despite the accumulation of research suggesting an association.

 In this study, we examined the association between the volume of basal ganglia and cardiorespiratory fitness levels in older adults. Given the extant rodent literature and one study in humans [21], we predicted that the size of the dorsal striatum, including the caudate nucleus and putamen, and the size of the ventral striatum (i.e., nucleus accumbens) would be positively associated with cardiorespiratory fitness compared to the output nucleus of this system, the globus pallidum, which has been less frequently associated with exercise. Furthermore, we predicted that any association between fitness and dorsal striatum volume would have implications on cognitive tasks that are supported by these structures. Specifically, cognitive flexibility and attentional control paradigms have been closely linked to the function of the dorsal striatum [21–23]. Therefore, using mediation modeling, we predicted that the volume of the dorsal striatum, but not the ventral striatum, would mediate the association between higher cardiorespiratory fitness levels and elevated performance on attentional control and task switching paradigms.

## 2. Methods

### 2.1. Participants

One hundred and seventy-nine older adults (109 females; 56 males) between 59 and 81 yrs of age (mean  age = 66.6 years; standard  deviation = 5.6 years) participated in this study. Participants were recruited for a 1-year randomized controlled trial examining the effect of aerobic training on brain and cognition. We report here results from the initial baseline MR session. Subjects were recruited through community advertisements and physician referrals and were screened for dementia by the revised and modified Mini-Mental Status Examination [24]. Participants were excluded from participation if they did not reach the required cutoff of 51 (high score of 57).

Additional inclusion criteria have been described elsewhere [5, 9, 25–27]. In brief, participants were required to be 60+ years of age during the trial, capable of performing physical exercise, successfully complete a graded exercise test, have normal or corrected-to-normal vision, absence of clinical depression (as measured by the Geriatric Depression Scale [28]), and a low-active lifestyle at time of baseline assessment. All participants have received a physician's clearance to engage in a maximal graded exercise test and signed an informed consent approved by the University of Illinois.

 In addition to these criteria, all participants met or surpassed criteria for participating in a magnetic resonance imaging (MRI) study including no previous head trauma, no previous head or neck surgery, no diagnosis of diabetes, no neuropsychiatric or neurological condition including brain tumors, and no metallic implants that could interfere with or cause injury due to the magnetic field.

The current study focused on participants that had both high-resolution MRI data and completed the cognitive assessments described below. All analyses were performed in a pairwise manner with the subjects who had relevant data points. The specific degrees of freedom are reported for each test.

### 2.2. Cardiorespiratory Assessment

Aerobic fitness (VO_2max_) was assessed by graded maximal exercise testing on a motor-driven treadmill. The participant walked at a speed slightly faster than their normal walking pace (approximately 30–100 m/min) with increasing grade increments of 2% every 2 min. A cardiologist and nurse continuously monitored measurements of oxygen uptake, heart rate and blood pressure. Oxygen uptake was measured from expired air samples taken at 30 s intervals until a maximal VO_2_ was attained or to the point of test termination due to symptom limitation and/or volitional exhaustion. VO_2max_ was defined as the highest recorded VO_2_ value when two of three criteria were satisfied: (1) a plateau in VO_2_ peak between two or more workloads; (2) a respiratory exchange ratio >1.00; (3) a heart rate equivalent to their age predicted maximum (i.e., 220-age).

### 2.3. Body Mass Index (BMI)

Height and weight were measured using a Seca electronic scale and stadiometer (model 763 1321139). Participants were measured while wearing light clothing and without shoes. BMI was calculated using the standard formula of weight (kg)/[height (m)]^2^.

### 2.4. MRI Acquisition and Volumetric Analysis

High-resolution T1-weighted brain images were acquired within two weeks of the cardiorespiratory fitness tests using a 3D Magnetization Prepared Rapid Gradient Echo Imaging (MPRAGE) protocol with 144 contiguous axial slices, collected in ascending fashion parallel to the anterior and posterior commissures, echo time (*TE*) = 3.87 ms, repetition time (*TR*) = 1800 ms, field of view (FOV) = 256 mm, acquisition matrix 192 mm × 192 mm, slice thickness = 1.3 mm, and flip angle = 8. All images were collected on a 3T head-only Siemens Allegra MRI scanner.

 For segmentation and volumetric analysis of the basal ganglia we employed FMRIB's Integrated Registration and Segmentation Tool (FIRST) in FMRIB's Software Library (FSL) version 4.1. FIRST is a semiautomated model-based subcortical segmentation tool utilizing a Bayesian framework from shape and appearance models obtained from manually segmented images from the Center for Morphometric Analysis, Massachusetts General Hospital, Boston, MA, USA. Structural and landmark information was obtained from 317 manually segmented and labeled T1 weighted images of the brain from normal children, adults, and pathological populations (including schizophrenia and Alzheimer's disease) and were modeled as a point distribution model in which the geometry and variation of the shape of the structure are submitted as priors. Volumetric labels are parameterized by a 3D deformation of a surface model based on multivariate Gaussian assumptions. FIRST then searches through linear combinations of shape modes of variation for the most probable shape given the intensity distribution in the T1-weighted image (see Patenaude et al. [29], for further description of this method).

**Figure 1:**
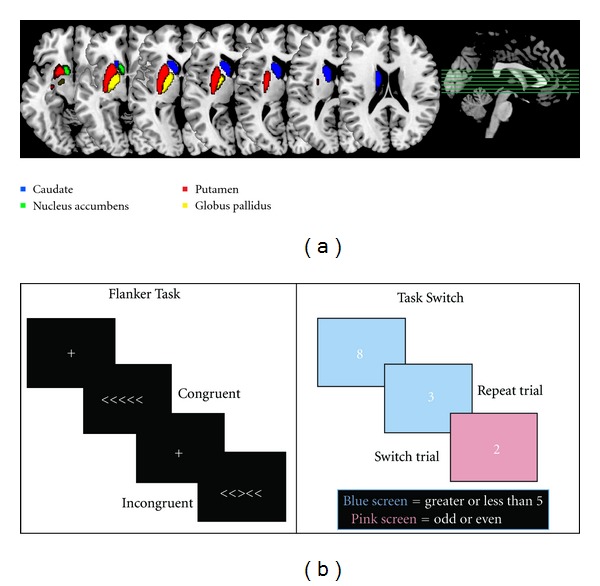
(a) Template maps for the four regions of interest used in this study. While only left hemisphere regions are shown here, data from both hemispheres was collapsed together in the final analyses. (b) Illustration of the two behavioral paradigms tested.

 This method first runs a two-stage affine registration to a standard space template (MNI space) with 1 mm resolution using 12 degrees of freedom and a subcortical mask to exclude voxels outside the subcortical regions. Second, the left and right basal ganglia including the caudate nucleus, putamen, pallidum, and nucleus accumbens are segmented with 30, 40, 40, and 50 modes of variation, respectively. The modes of variation are optimized based on leave-one-out cross-validation on the training set and increase the robustness and reliability of the results [29]. Finally, boundary correction takes place for each structure that classifies the boundary voxels as belonging to the structure or not based on a statistical probability (*z*-score > 3.00; *P* < 0.001).  Figure 1(a) shows the template masks for these nucleus volumes in the left hemisphere. Although volumes were estimated separately for the left and right hemispheres the volumetric estimates were highly correlated between the hemispheres for the nucleus accumbens (*r*(139) = 0.552, *P* < 0.001), caudate nucleus (*r*(151) = 0.638, *P* < 0.001), putamen (*r*(151) = 0.542, *P* < 0.001), and globus pallidus (*r*(151) = 0.806, *P* < 0.001). Therefore, a total volume estimate was generated for each nucleus by summing the values for the two hemispheres together.

 Intracranial volume (ICV) is frequently used to adjust the regional volumes for sex and height (e.g., [1]). Here, we calculated ICV as the sum of gray, white, and cerebrospinal fluid using FMRIB's automated segmentation tool in FSL version 4.1 [30, 31]. In accordance with other volumetric studies, ICV was used as a covariate in all analyses reported below [5, 9, 32].

### 2.5. Behavioral Tasks

Figure 1(b) shows the time line of each trial for the two behavioral paradigms utilized in this study. Results from the full battery of cognitive tasks have been reported in previous studies [5, 9, 25–27]. Given the *a priori *interest in the basal ganglia, we analyzed the flanker and task switching batteries because they both have been associated with basal ganglia function in animal and human studies. Cognitive tasks for which we did not predict *a priori* to be associated with the basal ganglia were not analyzed in this study.

#### 2.5.1. Task Switching

This task provided a measure of executive function by testing participants' abilities to flexibly switch their focus of attention between multiple task sets. In this task participants had to switch between judging whether a number (1, 2, 3, 4, 6, 7, 8, or 9) was odd or even and judging whether it was low or high (i.e., smaller or larger than 5). Numbers were presented individually for 1500 ms against a pink or blue background at the center of the screen, with the constraint that the same number did not appear twice in succession (see Figure 1(b)).

If the background was blue, participants used one hand to report as quickly as possible whether the letter was high (“*X*” key) or low (“*Z*” key). If the background was pink, participants used their other hand to report as quickly as possible whether the number was odd (“*N*” key) or even (“*M*” key). Participants completed four single task blocks (2 blocks of odd/even and 2 blocks of high/low) of 24 trials each. Due to the difficulty of this task, participants were provided with a practice block in which they switched from one task to the other for 120 trials. This practice block allowed participants to become acquainted with the switching block and ensured compliance with task instructions. Finally, they completed a switching block of 120 trials during which the task for each trial was chosen randomly.

For the current study, we examined local switch cost, which refers to the difference in performance for trials when the preceding trial involved the same task (nonswitch trial) and those when the preceding trial was of the other task (switch trial), and represents a measure of attentional set reconfiguration and inhibition, two subcomponents of executive function [33]. Reaction time measures (based on mean reaction time) were used for computing local switch cost. Consistent with prior studies using this paradigm [27], we used accuracy rates (percent correct) as an outcome measure and also calculated an accuracy cost score that reflects the difference in accuracy rates between switch and repeat conditions within the switching block.

#### 2.5.2. Flanker Task

A modified flanker paradigm required participants to identify the orientation of a central arrow cue that was flanked by arrows that were in either a congruent (e.g., >>>>>) or incongruent (e.g., >><>>) orientation. We used reaction times for both conditions in addition to proportional cost. Cost was calculated by subtracting the reaction times of congruent trials from those of incongruent trials and dividing by the reaction time of congruent trials. Such an approach accounts for individual differences in perceptual speed (see Figure 1(b)).

### 2.6. Data Analysis

#### 2.6.1. Partial Correlations

Initial analyses examined the distribution of the variables for skew using Q-Q plots. All variables were confirmed to be normally distributed by both visual inspection of the Q-Q plots and a resulting *r*-squared value to the simulated distribution of >0.95. Any missing values were eliminated from calculations. This resulted in degrees of freedom ranging from 139 to 178 in this sample, depending on the variables used in each analytical comparison. Bivariate analyses examined the correlation between VO_2max_ and age, sex, education, ICV, and BMI using Pearson's correlation coefficients. These variables were then used as covariates in all subsequent analyses looking at VO_2max_, task and brain volume relationships.

Once the covariate relationships were determined, partial correlations were then used to test the relationship between VO_2max_ and pallidum, putamen, nucleus accumbens, and caudate nucleus volumes. Next, the direct effect of VO_2max_ on the cognitive tasks described above was estimated. Partial correlations were then conducted to assess the association between basal ganglia volumes and cognitive tasks.

#### 2.6.2. Mediation Analysis

To determine indirect pathways, we conducted a bootstrapped mediation analysis. The main requirement for mediation is that the *indirect effect* of the independent variable (VO_2max_) through the mediator (e.g., caudate nucleus volume) on the dependent variable (e.g., task switch % accuracy) be significant.

Mediation analyses were conducted using the *indirect macro* designed for SPSS [34]. This macro uses bootstrapped sampling to estimate the indirect mediation effect of volume on the relationship between VO_2max_ and Flanker Task or task switching performance. In this analysis, 5,000 bootstrapped samples were drawn with replacement from the dataset to estimate a sampling distribution for the indirect mediation pathway. Indirect effects and 95% confidence intervals are reported. Mediation indirect effects can be interpreted as the strength of the relationship between the independent variable (VO_2max_) and dependent variable (e.g., Task Switching performance) when accounting for the mediating pathway [35]. We report adjusted *R* ^2^ values for an estimate of the effect size. Each outcome measure was used as a dependent variable in separate analyses. All models controlled for the variance from age, education, BMI, ICV, and sex.

## 3. Results

### 3.1. Covariate Effects

**Figure 2:**
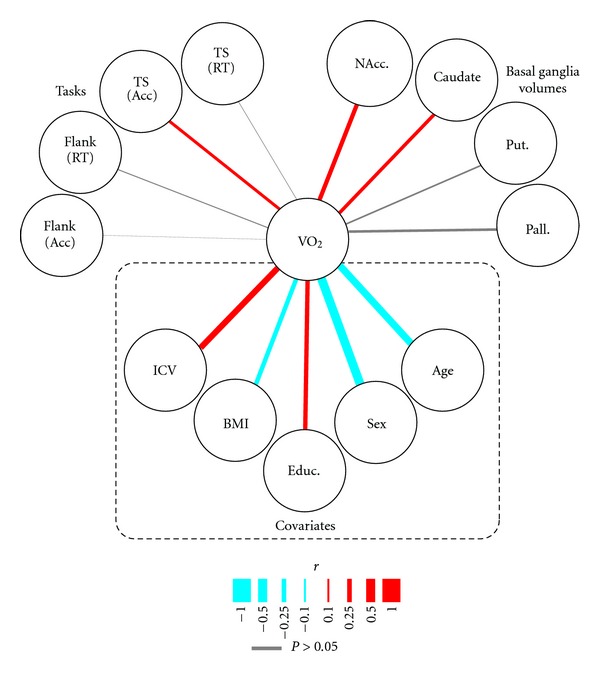
Correlation paths relating directly to measures of cardiorespiratory integrity (VO_2max_). Colored lines show significant correlations and the width of the line reflects the strength of the relationship. All correlation values are reported in the text. Variables highlighted in the “Covariates” section were used as control factors in the partial correlation analyses for the other pathways. Behavioral results reflect between condition changes in performance (i.e., costs). VO_2max_: VO_2_, Task Switching: TS, Flanker Task: Flank, accuracy: Acc, reaction time: RT, nucleus accumbens: NAcc., putamen: Put. globus pallidus: Pall. education: Educ., body mass index: BMI, intracranial volume: ICV.

We first examined the association between cardiorespiratory fitness and other factors that may act as sources of noise in the analyses. These relationships are illustrated in Figure 2 (Covariates section). As expected, there were significant negative correlations between VO_2max_ and BMI (*r*(178) = −0.242, *P* = 0.001), sex (logistic  beta = −1.034, *r*(178) = −0.480, *P* < 0.001), and age (*r*(178) = −0.391, *P* < 0.001). Thus, cardiorespiratory fitness levels were lower in overweight and obese subjects, lower in women than men, and lower in older aged participants. We also found positive correlations between VO_2max_ and years of education (*r*(178) = 0.234, *P* = 0.002) as well as intracranial volume (*r*(178) = 0.322, *P* < 0.001). Thus, cardiorespiratory fitness increased with educational attainment and overall body size. This latter association is presumably driven by the sex effects since men had significantly larger intracranial volumes than women (*t*(156) = 7.86, *P* < 0.001). These five factors were included in all subsequent analyses as covariates.

### 3.2. Associations between Cardiorespiratory Fitness and Task Performance

Next, we examined relationships between cardiorespiratory fitness and performance in the two behavioral tasks. These relationships are illustrated in Figure 2 (Tasks region). To get a full appreciation of relationships to the behavioral tasks, Table 1 shows the simple bivariate correlations between all demographic variables, cardiorespiratory fitness, and behavioral task performance. However, our core analysis centers on relationships specific to cardiorespiratory fitness, therefore we used a partial correlation analysis to control for noncardiorespiratory effects (see Section 2).

Unlike the results from previous studies [15, 21, 36, 37], we did not see an association between cardiorespiratory fitness and performance in the Flanker Task. After adjusting for covariate effects, there was no significant correlation between VO_2max_ scores and percent interference for reaction times in the Flanker Task (*r*(149) = 0.049, *P* = 0.546). We also failed to detect a significant association between VO_2max_ and response times on either the congruent (*r*(149) = 0.035, *P* = 0.669) or incongruent (*r*(149) = 0.001, *P* = 0.994) conditions. This effect does not appear to be due to a lack of sensitivity in the overall behavioral responses, as there was a significant group-level increase in response times to incongruent trials, compared to congruent trials (mean  percent  interference = 14.9%, std = 10.4, *t*(166) = 18.55, *P* < 0.001).

We also failed to detect a significant association between fitness levels and accuracy rates in the Flanker Task. After controlling for covariate effects, there was no significant correlation between VO_2max_ scores and differences in accuracy between the incongruent and congruent conditions (*r*(149) = 0.008, *P* = 0.925). As with response times, there was no significant association between VO_2max_ scores and response accuracy during either the congruent (*r*(149) = 0.053, *P* = 0.521) or incongruent conditions (*r*(149) = 0.041, *P* = 0.648). However, these null effects may be driven by a ceiling effect in accuracy rates in this sample (congruent: mean = 95.89%, std = 9.40%; incongruent: mean = 93.43%, 13.00%).

We also failed to detect significant associations between cardiorespiratory fitness and local switch costs in the Task Switching experiment. That is, after adjusting for covariate effects, VO_2max_ was not significantly correlated with the delayed reaction times that result from switching between trial types (*r*(150) = 0.025, *P* = 0.398). Although, there was a significant positive correlation between VO_2max_ and response times for the repeat condition such that individuals with higher VO_2max_ had slightly slower response times (*r*(150) = 0.312, *P* < 0.001). This correlation was not seen with response times during switch trials (*r*(150) = 0.019, *P* = 0.815). The lack of a correlation between fitness and switch costs does not appear to be due to a floor effect in the switching costs because across all subjects the average response times were significantly slower after a switch relative to a repeat condition (mean  difference = 530.5, std = 225.94, *t*(162) = 29.976, *P* < 0.001).

In contrast to response times, we did observe a small, but significant, association between cardiorespiratory fitness and accuracy in the Task Switching experiment. After adjusting for covariate effects, VO_2max_ scores were positively correlated with task-switch accuracy for switch trials after subtracting the accuracy rates from repeat trials (*r*(150) = 0.160, *P* = 0.048). In general, switching accuracies were negative (mean = −0.167, std = 0.228, *t*(165) = −9.42, *P* < 0.001) meaning that more errors occurred on switch trials compared to repeat trials. Within each condition used to calculate the accuracy cost, we also found a positive correlation between VO_2max_ scores and accuracy rates for repeat trials (*r*(150) = 0.176, *P* = 0.030) and switch trials (*r*(150) = 0.177, *P* = 0.029). Thus, individuals with higher VO_2max_ scores were both more accurate in general and were better able to maintain accurate responses immediately following a change in task goals.

#### 3.2.1. Associations between Cardiorespiratory Fitness and Basal Ganglia Volumes

We next set out to identify relationships between cardiorespiratory fitness and volumetric measures of the subcortical nuclei that compose the basal ganglia system. These relationships are illustrated in the “Basal Ganglia Volumes” region of Figure 2.

 Our first analysis focused on the nuclei that compose the major input of the basal ganglia system, the striatal nuclei. Partial correlations, after adjusting for covariate effects, revealed significant associations between cardiorespiratory fitness and the volume of nuclei that compose the caudal sections of the striatum. Specifically, VO_2max_ scores positively correlated with the volume of the nucleus accumbens (*r*(138) = 0.232, *P* = 0.006) and caudate nucleus (*r*(151) = 0.186, *P* = 0.022). In both cases, individuals with higher cardiorespiratory fitness also had larger volumes of these striatal nuclei. In contrast, we did not observe this same correlation with the volume of the putamen (*r*(151) = 0.085, *P* = 0.296).

 We next looked at the principal output nucleus of the basal ganglia, the globus pallidus. Although we found a trend for a positive association between VO_2max_ scores and pallidal volume, this relationship did not reach statistical significance (*r*(151) = 0.139, *P* = 0.086). Thus, although VO_2max_ had significant associations with sections of the input to the basal ganglia, the relationship between cardiorespiratory fitness and the globus pallidus remains uncertain.

#### 3.2.2. Associations between Basal Ganglia Volumes and Task Performance

**Figure 3:**
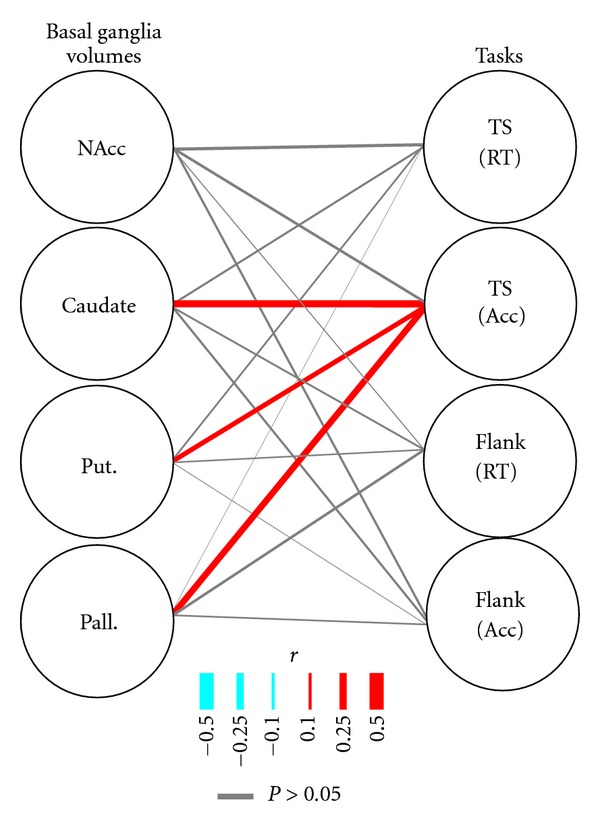
Correlation paths between basal ganglia regions of interest volume and behavioral costs for both tasks. Same plotting convention as Figure 2.

According to current methods of mediation analysis, detecting direct pathway effects (i.e., VO_2max_ to task performance) is not a necessary condition for indirect pathway effects (i.e., VO_2max_ to brain volume to task performance) [34]. However, in order to identify possible indirect mediating pathways between cardiorespiratory fitness and task performance through basal ganglia volumes, the direct pathways between brain volumes and behavior must also be established. These pathways are illustrated in Figure 3.

 For performance in the Flanker Task, we failed to observe any significant associations between basal ganglia volume and task performance. Specifically, the partial correlation analysis failed to detect a significant relationship between response interference in the Flanker Task and the volume of either the nucleus accumbens (*r*(138) = 0.031, *P* = 0.721), caudate nucleus (*r*(150) = 0.062, *P* = 0.446), putamen (*r*(150) = 0.131, *P* = 0.106), or globus pallidus (*r*(150) = 0.087, *P* = 0.284). There was also a lack of significant relationships between accuracy in the Flanker Task and the volume of either the nucleus accumbens (*r*(138) = 0.078, *P* = 0.361), caudate nucleus (*r*(149) = −0.074, *P* = 0.368), putamen (*r*(149) = 0.028, *P* = 0.732), or globus pallidus (*r*(149) = 0.054, *P* = 0.511). This same pattern was also present when the partial correlations were run against the congruent and incongruent scores in the Flanker Task separately (Table 2). Therefore, we can rule out any possible indirect pathway effects in the Flanker Task in our sample.

 Similarly, we failed to detect any significant associations between Task Switching costs in response times and basal ganglia volumes. The partial correlation analysis failed to detect a significant correlation between switching and the volume of the nucleus accumbens (*r*(138) = 0.102, *P* = 0.209), caudate nucleus (*r*(148) = −0.069, *P* = 0.398), the putamen (*r*(148) = 0.059, *P* = 0.477), or the globus pallidus (*r*(148) = −0.003, *P* = 0.973). We did detect a negative correlation between caudate volume and reaction times in the repeat trial condition (see Table 2), suggesting that greater caudate nucleus volume predicted overall faster reaction times in the simpler condition. However, the speed cost of changing between conditions was not predicted by caudate volume. Therefore, similar to performance in the Flanker Task, we can rule out the possibility of indirect mediating pathways between cardiorespiratory fitness and response times in the Task Switching experiment in our sample.

 We did, however, observe significant associations between the volume of basal ganglia nuclei and accuracy in the Task Switching experiment. While the volume of the nucleus accumbens failed to have a significant partial correlation (*r*(138) = 0.089, *P* = 0.293), we observed significant positive correlations between the volumes of the caudate nucleus (*r*(150) = 0.247, *P* = 0.002), the putamen (*r*(150) = 0.164, *P* = 0.044) and the globus pallidus (*r*(150) = 0.218, *P* = 0.007) with accuracy rates in the switch condition. When we look at the components of the accuracy cost score, we see that both repeat and switching trials were negatively correlated with the volume of the caudate nucleus and the globus pallidus, but not the putamen (see Table 2). Thus, these two nuclei had predictive value for both overall accuracy rates and accuracy costs in switching between conditions. Taken together, these results suggest that, in our sample, the size of the two largest striatal nuclei and the output nucleus of the basal ganglia were predictive of better accuracy during Task Switching conditions. This leaves open the possibility of indirect mediating pathways of the basal ganglia in the relationship between cardiorespiratory fitness and accuracy in the Task Switching experiment.

#### 3.2.3. Mediating Pathways

**Figure 4:**
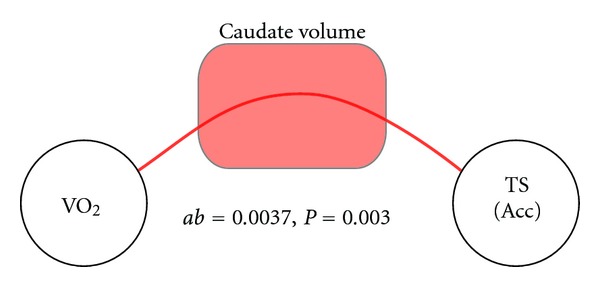
Indirect mediating pathway between cardiorespiratory fitness and Task Switching performance via the volume of the caudate nucleus.

The presence of significant relationships between (a) VO_2max_ and caudate nucleus volume and (b) caudate nucleus volume and accuracy in the Task Switching experiment, suggests that the caudate nucleus may be a candidate mediator for the relationship between cardiorespiratory fitness and task switch performance. We set out to determine the degree to which the caudate nucleus volume mediated the relationship between VO_2max_ and task performance. We tested this using a bootstrapped mediation analysis approach [34] and included all the covariate terms in the regression model. This analysis revealed a significant indirect mediation pathway between VO_2max_ and accuracy through caudate nucleus volume (Figure 4; *ab* coefficient = 0.0037, *P* = 0.003, 95% CI = 0.0009–0.0085, df = 150). The effect size of this mediation effect was small (*R* ^2^ = 0.1661), however including the caudate volume in the calculation of the direct pathway from VO_2max_ to Task Switching accuracy accounted for approximately 33% of the total effect (*c* coefficient = 0.0106, *c*′coefficient = 0.0070). Thus, caudate nucleus volume was a significant, partial mediator of the relationship between cardiorespiratory fitness and task performance.

## 4. Discussion

Our results show that similar to our previous findings in cortical regions [5, 9], increased cardiorespiratory fitness, that is, higher VO_2max_, in older adults is associated with larger volumes of several core basal ganglia nuclei, specifically the caudate nucleus and nucleus accumbens. The putamen and globus pallidus were not significantly associated with VO_2max_. Cardiorespiratory fitness levels were also positively associated with accuracy rates in a Task Switching paradigm that assesses cognitive flexibility. While several basal ganglia nuclei were positively associated with Task Switching accuracy, only the caudate nucleus met the assumptions necessary to perform subsequent mediation analyses. This subsequent analysis found that the caudate nucleus volume was a partial mediator of the relationship between fitness and Task Switching accuracy. These results highlight a key candidate pathway by which differences in cardiorespiratory fitness may regulate cognitive flexibility in older adults.

 Our results are consistent with previous studies demonstrating that dopamine receptor pathways in the nucleus accumbens and mesolimbic circuitry mediate the rewarding aspects of exercise in rodents [38–40]. In addition, rodents that are provided access to running wheels show attenuated effects of methamphetamine and cocaine in the nucleus accumbens [41–43] and increased gene expression in both the dorsal and ventral striatum for D1 and D2 receptors and associated G-proteins [40]. Exercise attenuates the neurotoxic effects of 6-hydroxydopamine on dopaminergic circuitry in the dorsal striatum [16, 44], possibly through BDNF pathways [44]. Hence, results from rodent studies of exercise are in line with our finding that cardiorespiratory fitness levels in humans are positively associated with the size of basal ganglia. The results from rodent models also provide potential molecular mechanisms by which fitness is associated with greater volume of the basal ganglia. Although the cellular and molecular correlates of MRI-based volumetric estimates remain unknown, our finding of larger volumes as a function of cardiorespiratory fitness is interesting in the light of this previous literature in rodents.

To the extent that cardiorespiratory fitness is modifiable by participation in regular aerobic exercise [5], our results suggest that age-related loss in volume of the caudate nucleus and nucleus accumbens may be remediable by exercise. In relation to this, however, a recent study by our group [5] reported that a randomized 1-year aerobic exercise intervention failed to significantly increase caudate nucleus volume, despite a significant increase in the size of the hippocampus. Hence, the results from the cross-sectional analysis reported here are not in line with the results from the randomized exercise intervention. There are several potential explanations for this apparent discrepancy. First, the sample size in the current study (*N* = 179) was larger than the results from the Erickson et al. [5] study (*N* = 60 per group) suggesting that the intervention might have been underpowered to detect exercise-induced changes in volume of the caudate nucleus. Such a conclusion is strengthened by a closer examination of the Erickson et al. [5] intervention results that showed a trend for the volume of the caudate nucleus to be different between the exercise and stretching groups at post-assessment, but the difference did not reach significance. Longer randomized trials with larger sample sizes are needed to confirm whether an exercise intervention is capable of increasing the size of the caudate nucleus and nucleus accumbens.

 A second explanation for the apparent discrepancy between our results and those of Erickson et al. [5] is the inherent limitation in all cross-sectional study designs. Specifically, it is possible that an uncontrolled third variable, correlated with both cardiorespiratory fitness and caudate nucleus volume, but not with exercise participation *per se*, explains the discrepancy between our results and those of Erickson et al. [5]. In fact, there is a genetic component to VO_2max_ [45] that might explain both the link to the volume of the basal ganglia and cognitive flexibility. Again, these results suggest that randomized trials with larger sample sizes and longer periods of an exercise treatment are necessary to disentangle the possible explanations for the discrepancy between the cross-sectional results reported here and the intervention results reported in Erickson et al. [5]. In fact, a larger scale intervention study could attempt to replicate the cross-sectional results that we describe here in addition to testing whether an exercise regimen could alter the size of the basal ganglia. Another method of testing these hypotheses would be to recruit individuals with dysfunction or deficits of the basal ganglia into an exercise intervention to examine whether volume could be altered in these at-risk or impaired populations.

 To our knowledge, there is only one other cross-sectional neuroimaging study examining the volume of the basal ganglia in relation to cardiorespiratory fitness levels [21]. In that study of 55 preadolescent children (9-10 years old) higher fitness levels were associated with greater volume of the caudate nucleus, putamen, and globus pallidus, but not the nucleus accumbens. Interestingly, the volume of the putamen and globus pallidus was also correlated with performance on a Flanker Task. The results we report here are clearly only partially consistent with the results from Chaddock et al. [21]. First, we failed to find any associations with Flanker Task performance and the size of the basal ganglia, and second, we only find associations with fitness for the caudate nucleus and nucleus accumbens, not the putamen or pallidum. One possible explanation for the inconsistency between our study and that of Chaddock et al. [21] is that there are maturational differences in the growth and later atrophy of the basal ganglia [46] such that fitness effects emerge on different brain structures at different time points throughout the lifespan. In fact, such an explanation is likely given developmental trajectories of brain growth, pruning, and myelination [47] earlier in development in contrast to atrophy and volumetric loss late in life [2]. Of course another possible explanation for these between-study differences could simply result from the inter-subject variability inherent in all cross-sectional experimental designs.

 Here we were able to demonstrate that greater volume of the caudate nucleus is a significant indirect pathway between cardiorespiratory fitness and cognitive flexibility as assessed by a Task Switching paradigm. Switching abilities and reversal learning are supported by the basal ganglia, medial frontal cortex, and dorsolateral prefrontal cortex in both rodents and humans [48, 49]. Our results, therefore, are consistent with this literature and suggest that variation in cardiorespiratory fitness levels might explain significant individual variability in correlations between volume and task-switch performance. Interestingly, we failed to find associations with performance on the Flanker Task, despite previous studies showing links with attentional control and function of the basal ganglia circuits [21, 50]. This dissociation between the task-switch and flanker paradigms suggests some specificity of the function of the basal ganglia circuits in late adulthood. One explanation might be that tasks involving attentional control and response conflict, like the flanker paradigm, might be more dependent on prefrontal structures than basal ganglia in late life. This hypothesis is supported by a recent study demonstrating that older adults had less activity in the dorsal striatum during performance of a Stroop task compared to a younger group [50]. Given the presence of cortico-striatum loops, it is likely that a distributed circuit of frontal, parietal, and striatum is involved in mediating both attentional and switching behaviors, but that the relative importance of these structures changes with age.

In addition, we failed to find significant associations between Flanker Task performance and fitness levels despite numerous other studies finding significant associations throughout the lifespan [15, 21, 36, 37, 51–53]. It is likely that our limited range of cardiorespiratory fitness levels precluded our ability to detect significant associations and that the correlations with Task Switching performance are more robustly associated with fitness levels. In any case, it will be important for future studies with a wider range of fitness levels to examine the links between Flanker performance and caudate nucleus volume.

Finally, as alluded to earlier, there are several limitations to the current study that warrant further exploration in future work. First, our study was cross-sectional in nature so causal conclusions about how increased cardiorespiratory fitness might influence the size of the basal ganglia are inherently limited. It will be critical for randomized exercise interventions conducted with a large well-characterized cohort to determine whether it is possible to alter the size of these structures. Second, we did not have any tasks (i.e., reward-based) that might be considered to be dependent on the nucleus accumbens, so we were unable to examine the behavioral importance of greater nucleus accumbens volume with higher fitness levels. However, despite these limitations we were able to detect associations using a relatively large and homogeneous sample of older adults and cognitive tasks that have been previously linked to the function of the dorsal striatum. In sum, we find that cardiorespiratory fitness levels are positively associated with volume of the caudate nucleus and nucleus accumbens in late adulthood and that the size of the caudate nucleus is a significant mediator between fitness and performance in the Task Switch paradigm.

## Figures and Tables

**Table 1:** Simple bivariate Pearson correlations between demographic variables, cardiorespiratory fitness, and behavioral data. ^∗^Significant at *P* < .05; ^∗∗^significant at *P* < .01.

	VO_2_	Age	Sex	Education	BMI
Flanker					
Con RT	−.178^∗^	.283^∗∗^	.207^∗∗^	.009	−.036
Inc RT	−.144	.162^∗^	.211^∗∗^	.042	−.056
Cost (RT)	.020	−.179^∗^	.064	.023	−.034
Con Acc	.199^∗∗^	−.277^∗∗^	−.133	.092	.060
Inc Acc	.112	−.168^∗^	−.022	.052	.009
Cost (Acc)	−.055	.099	.084	−.015	−.060
Task-switch					
Repeat RT	−.021	.006	.135	−.034	.005
Switch RT	−.082	−.011	.173^∗^	−.105	.020
Cost (RT)	−.071	−.004	.151	−.088	.025
Repeat Acc	.174^∗^	−.224^∗∗^	.001	.040	.135
Switch Acc	.231^∗∗^	−.270^∗∗^	−.032	.061	.130
Cost (Acc)	.190^∗^	−.281^∗∗^	−.027	.022	.154

**Table 2:** Partial correlations between nuclear volumes and components of the behavioral scores.

	Nucleus accumbens	Caudate nucleus	Putamen	Pallidum
Flanker				
Con RT	−.114	−.015	−.054	.013
Inc RT	−.096	−.037	−.127	−.035
Cost (RT)	.031	.062	.131	.087
Con Acc	.132	.122	.045	−.034
Inc Acc	.176	.013	.069	−.012
Cost (Acc)	.078	−.074	.028	.054
Task-switch				
Repeat RT	.010	−.238^∗∗^	−.018	.089
Switch RT	.079	−.103	.081	.016
Cost (RT)	.107	−.069	.058	−.003
Repeat Acc	.120	.286^∗∗^	.115	.176^∗^
Switch Acc	.133	.274^∗∗^	.153	.217^∗∗^
Cost (Acc)	.089	.247^∗∗^	.164^∗^	.218^∗∗^
